# Evaluation of polybenzimidazole-based polymers for the removal of uranium, thorium and palladium from aqueous medium

**DOI:** 10.1098/rsos.171701

**Published:** 2018-06-20

**Authors:** V. Vijaya Kumar, C. Ramesh Kumar, A. Suresh, S. Jayalakshmi, U. Kamachi Mudali, N. Sivaraman

**Affiliations:** 1 Organic Chemistry Laboratory, Department of Chemistry, Vel Tech Rangarajan Dr Sagunthala R&D Institute of Science and Technology, Avadi, Chennai 600 062, Tamil Nadu, India; 2 Indira Gandhi Centre for Atomic Research, Homi Bhabha National Institute, Kalpakkam 603102, India

**Keywords:** polybenzimidazole-based polymer resin, column chromatography, uranium, thorium and palladium

## Abstract

Four types of polybenzimidazole (PBI)-based polymers (*m*-PBI, *p*-PBI, pyridine-based *m*-PBI and alkylated *m*-PBI) have been prepared and characterized. Extraction behaviour of heavy metal ions, *viz.* U(VI), Th(IV) and Pd(II), with these polymers was investigated. Distribution ratios for the extraction of these metal ions were measured as a function of nitric acid concentration. Extraction data reveal that, in general, *p*-PBI exhibits a higher distribution ratio for U(VI), Th(IV) and Pd(II) compared with the other polymeric resins evaluated in the present study. Column chromatography experiments were carried out with a solution of U(VI), Th(IV) and Pd(II) in dilute nitric acid media using columns packed with *m*- and *p*-PBI polymeric material for understanding the sorption and elution behaviour. The *p*-PBI-based resin has shown higher palladium sorption capacity (1.8 mmol g^−1^). The studies also established that *p*-PBI resin is a potential candidate material for the recovery of U(VI) and Th(IV) (capacity 0.22 mmol g^−1^ and 0.13 mmol g^−1^) from an aqueous stream, e.g. mine water samples.

## 1. Introduction

Separation technologies are being employed for the recovery of metal ions at various stages of the nuclear fuel cycle [1]. Several solvent extraction and chromatography-based processes have been proposed for the recovery of radionuclides from an aqueous stream. Chromatography methods are generally preferred if the recovery of metal ions is from a dilute aqueous stream. Extraction chromatography is an important separation method and is extensively used in analytical chemistry. This method is based on the extraction of metal ions by organic extractants impregnated in a solid support. Uptake of minor actinides and lanthanides from nitric acid medium by dihexyl-*N*,*N*-diethylcarbamoylmethyl phosphonate (DHDECMP) by extraction chromatography has been reported [2–4]. Regarding actinides, use of trialkyl phosphine oxide functionalized task specific ionic liquids for the recovery of actinides [5] and separation of minor actinides from acidic PUREX waste solution using bis(2-ethylhexyl) sulfoxides have also been reported [6]. Mohapatra *et al.* [7] investigated the uptake of actinides using diglycolamide-grafted resins as stationary phase. Several studies also reported recovery of lanthanides and actinides using octyl(phenyl)-*N*,*N*-diisobutylcarbamoylmethylphosphine oxide (CMPO) as the stationary phase [8–13]. Suresh *et al.* [14] have compared the extraction behaviour of CMPO-impregnated XAD-4 and XAD-7 resins for the extraction of uranium and neodymium. Sengupta *et al.* [15] studied the extraction of Am(III) using tripodal diglycolamide ligand in ionic liquids at room temperature for radioactive waste processing.

Ion-exchange chromatography, which is considered to be superior to extraction chromatography, has been widely employed in various stages of the nuclear fuel cycle [16] such us uranium milling; removal of contaminants from the primary coolant circuits, condensates and fuel storage pond water in pressurized water reactors; recovery of plutonium and transuranic and isotope protection. In addition, it has been used in the back end of the nuclear fuel cycle such as purification of plutonium, treatment of radioactive waste, scrap recovery and concentration of waste solutions. Acher *et al.* [17] studied plutonium absorption in nitric acid medium. Marsh *et al*. [18] studied the bifunctional anion-exchange resin for faster and quantitative sorption of plutonium. Sabharwal *et al*. [19] and Venkatesan *et al.* [20] have employed macroporous bifunctional phosphinic acid (MPBPA) resin for the recovery of U(VI) and Th(IV). da Silva Queiroz *et al.* [21] reported the separation of neodymium acetate from rare-earth carbonates using strong cation-exchange resin.

In the past few decades, platinum group metals (PGMs) have been widely used in various industrial applications, including automobiles, due to their specific physical and chemical properties [22–25]. Considering the ever-increasing worldwide demands and toxic effects of PGMs, it is essential to recover PGMs from waste media prior to discharge. High-level waste (HLW) solutions generated from processing of spent nuclear fuels, especially fast reactor fuels, also contain significant quantities of palladium. Various techniques based on solvent extraction, adsorption, chemical precipitation, membrane separation, ion-exchange and solvent extraction-based methods can be employed for its recovery [26–28]. Among the hydrometallurgical technologies, solvent extraction and adsorption have been widely used for the separation of PGMs [29] owing to their low cost and ease of operation. The selective recovery of Pd(II) from highly acidic solution using ion-imprinted chitosan fibres was reported [30]. However, elution of Pd from the weak-base resin was found to be difficult. Sorption of precious metal ions such as copper, silver, gold, mercury, palladium and platinum using *m*-PBI protonated with mercaptoacetic acid has been studied in acetate, carbonate and hydrochloric acid media. Chanda *et al*. have investigated the sorption of uranium from seawater in acetate and carbonate medium, and palladium in hydrochloric acid medium using *m*-PBI and its immobilized resins. However, immobilized *m*-PBI with DMG has shown higher extraction capacity than unmodified *m*-PBI [31–34]. Jiang *et al.* [35] investigated uptake of heavy metals such as copper and lead using polyaniline-based microspheres. Tang *et al.* [36] reported removal of heavy metals with sequential sludge washing techniques using saponin. Liquid-core capsules with a non-cross-linked alginate fluid core surrounded by a gallon membrane were prepared in single step and investigated for the sorption of heavy metal ions [37]. Korolev *et al*. [38] compared two conventional macroporous anion exchangers, namely Dowex21 K and Amberlite IRA-900, with strong-base, macroporous vinylpyridine resin VP1-AP for the recovery of Pd from nitric acid medium. Zhang *et al*. [39] studied the recovery of palladium from simulated nuclear waste using a macroporous vinylpyridine anion-exchange resin embedded in porous silica beads and reported that the weak-base resin (SiPyR-N3) shows stronger affinity towards Pd(II) than a strong-base (SiPyR-N4) anion exchanger. Biomass of *Escherichia coli* is capable of selective binding of Pd(II) in the Pd(II)–Pt(IV) bimetal solution [40]. Venkatesan *et al*. [41] investigated the uptake of palladium (20 mg g^−1^) from 3 to 4 M nitric acid medium from nuclear waste solution using Tulsion CH-95-attached PV-DVP.

Polybenzimidazole (PBI), a class of heterocyclic polymers, is being used as high-temperature proton exchange membranes for fuel cell applications including back-up power units, combined heat and power systems, and microportable power applications. PBI has unique physical, thermal and electrical properties, combined with good chemical resistance. As this polymer has benzimidazole groups which are more basic than pyridine, it is interesting to study its sorption behaviour with various heavy metal ions [42].

In the present study, PBI-based polymers were employed for the recovery of metal ions such as uranium, thorium and palladium from dilute nitric acid medium. The *m*-PBI polymeric resin was prepared by the condensation of 3,3′,4,4′-tetraminobiphenyl (TAB) with isophthalic acid in polyphosphoric acid as the polymerization solvent [43]. The *p*-PBI polymer was prepared to examine the influence of polymer linkage position on metal sorption. The role of imidazole N–H in the metal ion extraction was examined through preparation of alkylated polymer from the substituted monomer. Studies with pyridine-based *m*-PBI were also taken up in the present study to understand the role of the nitrogen-bearing benzene ring in the polymer matrix on the extraction of these metal ions. In this connection, four polymeric materials were prepared and characterized, and their extraction behaviour was evaluated with U(VI), Th(IV) and Pd(II) from nitric acid media. The study includes batch extraction, kinetics of sorption, static capacity, column chromatography studies with heavy metal ion solutions and elution characteristics of metal ions from loaded columns.

## 2. Experimental

### 2.1. Materials

All the chemicals used in the present work were of AR or GR grade (Loba Chemicals) and used without further purification. Toluene and triethylamine (Avra Chemicals) were used in the present study after purification by the standard distillation procedure. Uranium nitrate supplied by nuclear fuel complex (NFC), Hyderabad, thorium nitrate (Indian Rare Earths Limited (IREL), Udyogamandal, Kerala, India) and palladium nitrate (Aldrich) were used as such without further purification.

### 2.2. Preparation and characterization of polybenzimidazole polymers

#### 2.2.1. Preparation of *m*-polybenzimidazole (4) and *p*-polybenzimidazole (5) polymers

**Scheme 1.**
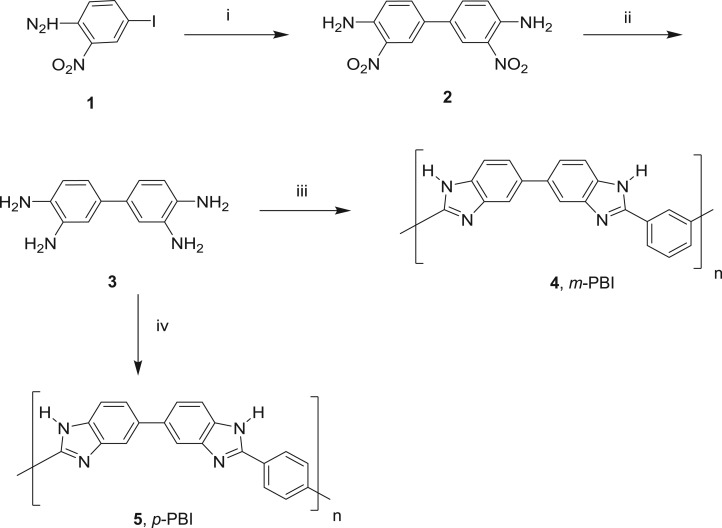
Reagents used and conditions adopted for the preparation of *m*-PBI and *p*-PBI: (i) Pd(OAc)_2_, Et_3_N, toluene, 110°C, 12 h, 89%; (ii) Sn, HCl, 25–40°C, 2 h, 73%; (iii) isophthalic acid, polyphosphoric acid, 120–220°C, 19–20 h and (iv) terephthalic acid, polyphosphoric acid, 120–220°C, 19–20 h.

Both *m*-PBI and *p*-PBI polymers were prepared [43] by the reactions shown in scheme 1 and their monomer characterization data are shown in electronic supplementary material, table S1. The first step involves the preparation of 3,3′-dinitro-4,4′-diaminobiphenyl (2) by the biaryl homocoupling of 4-iodo-2-nitroaniline (1) using palladium acetate in the presence of triethylamine in toluene. Palladium acetate (0.012 g, 0.1 mol%) was added to a mixture of 4-iodo-2-nitroaniline (1) (13.2 g, 50 mmol) and triethylamine (10.12 g, 100 mmol) in toluene (60 ml) with stirring at 25°C. The reaction mixture was heated in an oil bath at 110°C for 15 h and was then allowed to cool to room temperature. The product 3,3′-dinitrobenzene (DNB) was separated from the solvent by filtration. The crude biphenyl was washed with petroleum ether followed by dichloromethane to obtain 3,3′-dinitro-4,4′-diaminobiphenyl (2). As 3,3′-dinitro-4,4′-diaminobiphenyl is insoluble in toluene, it precipitated out with 89% yield; its melting point is 285°C.

Subsequently, the nitro groups were reduced to get the corresponding amines using Sn and HCl. Concentrated HCl (12 M; 50 ml) was added to a mixture of Sn metal powder (4.75 g, 40 mmol) and 3,3′-dinitro-4,4′-diaminobiphenyl (2.74 g, 10 mmol) with stirring at 25°C, and the temperature was raised to 40°C and the reaction mixture was further stirred for approximately 2 h at 40°C to ensure the complete reduction. The hydrochloride salt of tetramine was precipitated out and it was made alkaline with cold 20% NaOH solution. Free amine was filtered, washed with water and dried under vacuum to get 3,3′-diaminobenzidine (3); the yield was found to be 73% with 100% purity (see electronic supplementary material, figure S1); its melting point is 175–176°C.

In the last step, *m*-PBI (4) and *p*-PBI (5) were prepared by the polymerization of tetramine (3) with isophthalic acid or terephthalic acid, respectively, in the presence of polyphosphoric acid. In the case of *m*-PBI, polyphosphoric acid (36 g) was taken in a three-necked round-bottomed flask equipped with an overhead mechanical stirrer and heated at 120°C with a continuous flow of dry nitrogen. Tetraminobiphenyl (2.57 g, 12 mmol) was added at the same temperature and the reaction mixture was heated to 145°C with continuous stirring. Isophthalic acid (2.49 g, 15 mmol) was added at 145°C and the temperature was increased to 170°C. After stirring for approximately 5 h, the temperature was further increased to 220°C and this temperature was maintained for approximately 19 h. Subsequently, it was cooled to 120°C and slowly transferred to cold distilled water with vigorous stirring to get the polymer powder. The polymer was washed with saturated ammonium chloride solution followed by saturated sodium bicarbonate solution and the polymer was dried at 80°C in an oven for approximately 6 h to get 4.42 g of pure *m*-PBI (4). *p*-PBI (5) was prepared in a similar manner as described above using terephthalic acid.

#### 2.2.2. Preparation of alkylated *m*-polybenzimidazole (8)

**Scheme 2.**
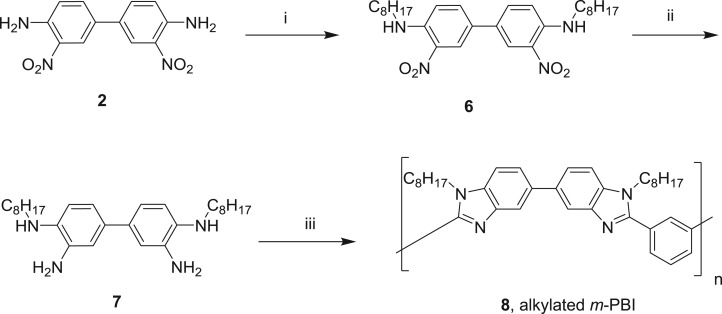
Reagents used and conditions adopted for the preparation of alkylated *m*-PBI: (i) C_8_H_17_Br, NaH, DMF, 7 h, 85%; (ii) Sn, HCl, 25–40°C, 2 h, 88%; (iii) isophthalic acid, polyphosphoric acid, 120–220°C, 19–20 h.

Polymerization of 3,3′-dioctylaminobenzidine (7) (see electronic supplementary material, for its preparation and characterization) with isophthalic acid in the polyphosphoric acid medium at 220°C afforded alkylated *m*-PBI with good yield (scheme 2). Polyphosphoric acid (36 g) was taken in a three-necked round-bottom flask equipped with an overhead mechanical stirrer and heated at 120°C with a continuous flow of dry nitrogen. Alkylated tetraminobiphenyl (5.26 g, 12 mmol) was added at the same temperature and the reaction mixture was heated to 145°C with continuous stirring. Isophthalic acid (2.49 g, 15 mmol) was added at 145°C and the temperature was raised to 170°C. After stirring for approximately 5 h, the reaction temperature was further increased to 220°C and this temperature was maintained for 19 h; then it was cooled to 120°C and added slowly to cold distilled water with vigorous stirring to get the polymer powder. The solid was washed with saturated ammonium chloride solution followed by saturated sodium bicarbonate solution, and the polymer was dried at 80°C in an oven for 6 h to get 6.2 g of alkylated *m*-PBI (8).

#### 2.2.3. Preparation of pyridine-based *m*-polybenzimidazole (9)

**Scheme 3.**
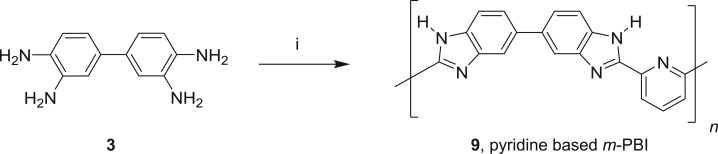
(i) 2,6-Pyridinedicarboxylic acid, polyphosphoric acid, 120–220°C, 19–20 h.

Pyridine-based PBI polymer (9) was prepared by polymerization of the monomer (3) with pyridine dicarboxylic acid in the polyphosphoric acid medium (scheme 3). Polyphosphoric acid (36 g) was taken in a three-necked round-bottom flask equipped with an overhead mechanical stirrer and heated at 120°C with a continuous flow of dry nitrogen. Tetra-aminobiphenyl (2.57 g, 12 mmol) was added at the same temperature and the reaction mixture was heated to 145°C with continuous stirring. 2,6-Pyridine dicarboxylic acid (2.49 g, 15 mmol) was added and the temperature was increased to 170°C. After stirring for approximately 5 h, the reaction temperature was increased to 220°C and this temperature was maintained for 19 h. Then, it was cooled to 120°C and slowly added to cold distilled water with vigorous stirring to get the polymer powder. The solid was washed with saturated ammonium chloride solution followed by saturated sodium bicarbonate solution, and the polymer powder was dried at 80°C in an oven for approximately 6 h to obtain 4.27 g of pyridine-based *m*-PBI (9).

### 2.3. Preparation of feed solutions for experiments

#### 2.3.1. Preparation of feed solutions of U(VI) in nitric acid for *D-*value measurements

Feed solution of uranium (600 µg ml^−1^) in various nitric acid concentrations was prepared from a standard solution with appropriate dilution. Free acidity and concentration of U(VI) of these solutions were ascertained by suitable analytical methods and these solutions were used for the measurement of distribution ratios by batch extraction. The uranium feed solution prepared in 0.01 M nitric acid medium was employed for kinetics experiments. Similarly, thorium (400 µg ml^−1^) and palladium (2000 µg ml^−1^) feed solutions were prepared in nitric acid medium for the distribution ratio studies and kinetics experiments. The concentrations of Pd(II) were kept higher to get a measurable concentration in the equilibrium phase after equilibration. Feed solutions of U(VI) of 100 µg ml^−1^ in 0.1 and 2 M nitric acid medium, and thorium nitrate feed solution of 100 µg ml^−1^ in 0.1 M nitric acid medium and palladium 200 µg ml^−1^ in 0.1 M HNO_3_ medium were employed for the column chromatography studies.

### 2.4. Measurement of distribution ratio

Distribution ratios (*K*_d_) for the extraction of U(VI), Th(IV) and Pd(II) with PBI polymeric resins were measured as a function of equilibrium aqueous phase nitric acid concentration at room temperature. The procedure involves the equilibration of approximately 100 mg of dried polymer material with 5 ml of the feed solution in an appropriate nitric acid medium in an equilibration tube for approximately 6 h at room temperature. Subsequently, the aqueous solution containing the metal-loaded polymer was centrifuged and the metal concentration in the aqueous phase was estimated. The distribution ratio was calculated using the following equation [4]:

2.1 $$K_{d}=\frac{C_{o}-C}{C}\times \frac{V}{W}\text{ml}\;{\text{g}}^{-1}.$$

where *C*_o_ and C represent the metal ion concentrations in the aqueous phase before and after the equilibration, respectively, *V* is the volume (in ml) of the aqueous phase taken and *W* is the weight (in g) of the PBI polymeric resin used for extraction.

### 2.5. Stripping studies

Initially, batch studies were performed to identify a suitable stripping agent for the removal of metal ions from the metal-loaded polymeric material. It was observed that U(VI) and Th(IV) from the loaded polymers can be stripped with 5% ammonium carbonate and 6% sodium carbonate solution, respectively. A solution of acidic thiourea (1 M thiourea in 0.1 M HNO_3_) was employed for the stripping of Pd(II) from metal-loaded polymeric resin. The percentage of stripping was calculated using the following equation (see electronic supplementary material, table S3):

2.2 $$\%\;\text{stripping}=\frac{\text{concentration of metals stripped from resin}}{\text{initial concentration adsorbed by resin}}\times \text{100}.$$

### 2.6. Column studies with solutions of U(VI), Th(IV) and Pd(II)

Column experiments for uranium and thorium loading and their elution behaviour were carried out with *m*-PBI and *p*-PBI polymeric resins at room temperature. The polymeric resin was packed in a column (1 cm diameter and 10 cm height, and 6.5 cm bed length) with 6.5 g of *m*-PBI resin. The column was initially conditioned by passing 50 ml of 0.1 M HNO_3_. The feed solution was passed through the column at a flow rate of 0.5 ml min^−1^. The effluent samples were collected at regular intervals in a 10 ml volumetric flask and analysed by UV–Vis spectrophotometry using Arsenazo-III as the chromogenic agent. When the column capacity attained 100% breakthrough, the loaded resin was washed with 25 ml of 0.1 M HNO_3_ followed by 20 ml of Millipore water. Subsequently, elution of metal ions was carried out with 5% ammonium carbonate solution for U(VI) and 5% sodium carbonate solution for Th(IV). The eluted samples were collected in 5 ml volumetric flasks and the metal concentrations were analysed. Experiments were also carried out with *p*-PBI under identical conditions. Column experiments for loading and elution of U(VI) from 2 M nitric acid medium were also carried out.

Column experiments on loading and elution behaviour of Pd(II) were carried out with *p*-PBI polymeric resins (6.5 g) packed in a column (1 cm diameter and 10 cm height). Loading studies were carried out with palladium nitrate solution (200 µg Pd ml^−1^) in 0.1 M nitric acid medium. After achieving the 100% breakthrough, the column was washed with approximately 25 ml of 0.1 M HNO_3_. Palladium was eluted using a solution of thiourea (1 M in 0.1 M HNO_3_ medium). The eluted samples were collected in 10 ml volumetric flasks and palladium concentration was analysed.

### 2.7. Analytical procedures

Concentrations of U(VI) and Th(IV) in all samples were estimated by spectrophotometry using Arsenazo-III as the chromogenic agent at 655 and 661 nm for uranium and thorium, respectively. Palladium was determined by spectrophotometry using Arsenazo-III as the chromogenic agent at 628 nm. In studies involving Pd(II) elution with thiourea solution (1 M thiourea solution in 0.1 M nitric acid medium), the Pd–thiourea coloured complex was directly monitored spectrophotometrically at 317 nm. Free acidity was estimated by acid–base titration using standard NaOH solution with phenolphthalein as the indicator.

### 2.8. Adsorption equilibrium

To optimize the design of an adsorption system to remove solute from solutions, it is important to establish the most appropriate correlation for the equilibrium curve. There are several isotherm equations available for analysing experimental adsorption equilibrium data: the Freundlich, Langmuir, Temkin, Toth, Redlich–Peterson, Sips, Langmuir–Freundlich, B.E.T and Dubinin–Radushkevich. However, the Langmuir isotherm is considered to be the best among the two most common types of isotherms, *viz*. Langmuir and the Freundlich models. Therefore, we have investigated *p*-PBI resin for uranium sorption (concentrations varying from 400 to 2000 mg l^−1^ in 0.1 M HNO_3_ for 100 mg of *p*-PBI resin). Similarly, Th(IV) and Pd(II) adsorption isotherms were examined.

## 3. Results and discussion

### 3.1. Characterization of polymers

#### 3.1.1. Measurement of surface area and average pore size

The surface area and pore size of these polymers were determined using SURFER (Thermo Fisher Scientific) and the results are summarized in table 1. The relatively higher surface area and pore diameter facilitate the diffusion of metal ions into the polymer matrix and provide more accessible sites. Besides these factors, the geometry of metal complexes and the nature of metal ions influence uptake capacity, kinetics of sorption and distribution ratios. The results of elemental analysis of these polymers are summarized in table 2.

**Table 1.** Pore size and surface area of PBI-based polymers (BET).

polymer	average pore diameter (nm)	specific surface area (m^2^ g^−1^)
*m*-PBI	89	2
*p*-PBI	87	3
alkylated *m-*PBI	60	8
pyridine-based *m*-PBI	109	1

**Table 2.** Elemental composition of these resins. Theoretical values given in the parenthesis.

		elements (wt%)		
s. no	polymers name	C	H	N
1	*m*-PBI	78 (78.32)	5.9 (5.08)	15.4 (16.61)
2	*p*-PBI	76 (78.38)	6.3 (5.72)	17 (15.90)
3	pyridine-based *m*-PBI	72.1 (74.77)	6.1 (5.42)	22 (19.82)
4	alkylated *m*-PBI	81 (81.20)	8.8 (9.09)	9.6 (9.71)

#### 3.1.2. Thermogravimetric analysis

**Figure 1.**
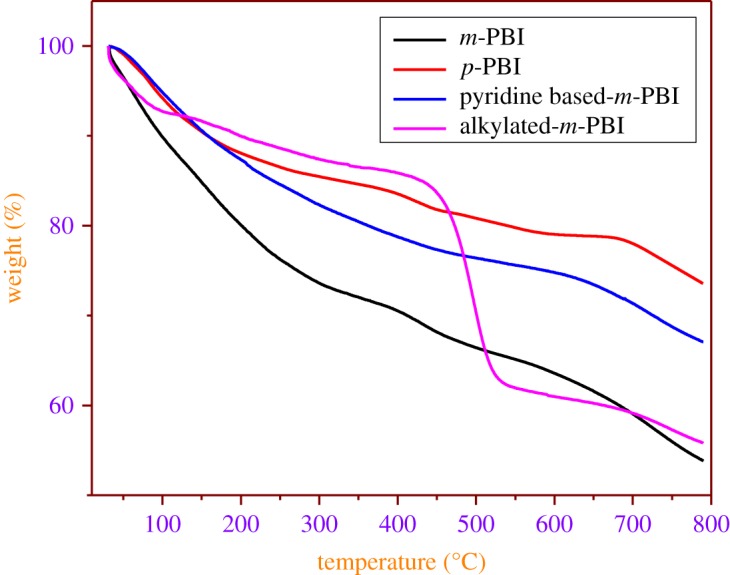
TGA of prepared PBI polymeric resins.

Figure 1 shows thermogravimetric analysis (TGA) of the prepared PBI polymeric resins such as *m*-PBI, *p*-PBI, pyridine-based *m*-PBI and alkylated *m*-PBI resins, which were annealed in a nitrogen environment (25–800°C) at the rate of 5°C for min^−1^. The initial significant weight loss from 30 to 130°C could be associated with the evaporation of water which is trapped in the pores of polymers. In the case of *m*-PBI, the middle region weight loss is higher than other resins, which may be attributed to the residue of polyphosphoric acid in the porous structure. In general, PBI-based polymers offer high thermal stability. Comparison of thermal stability of these polymers indicates that *p*-PBI is more stable than pyridine-based *m*-PBI, which in turn is more than *m*-PBI. The study indicated that decomposition takes place at approximately 400°C and 600°C for *m*-PBI and pyridine-based *m*-PBI, respectively, whereas, *p*-PBI is stable upto 800°C. Alkylated *m*-PBI lost more than 50% of its weight after 450°C. The high thermal stability of these polymeric resins can be attributed to the combined effect of an aromatic ring in the molecular structure and the hydrogen bonding of NH proton with the imidazole nitrogen lone pair of electrons in the polymer matrix.

#### 3.1.3. FTIR analysis

**Figure 2.**
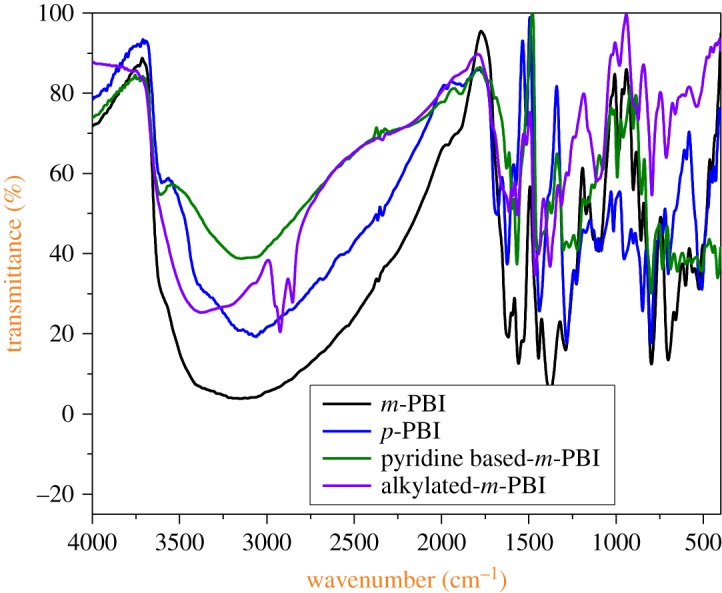
FTIR analysis of prepared PBI polymeric resins.

Figure 2 shows IR spectra of *m*-PBI, *p*-PBI, pyridine-based *m*-PBI and alkylated *m*-PBI. Spectra show strong absorptions in the benzimidazole ring region (1438–1631 cm^−1^), which is a characteristic absorption of in-plane vibration and combined C=C/C=N ring vibrations. The broad peak absorption from 2800 to 3500 cm^−1^ is attributed to N–H stretching vibration for *m*-PBI, *p*-PBI and pyridine-based *m*-PBI. However, the absence of a broad peak in the above-mentioned region and the peaks at 2800–2900 cm^−1^ for alkylated *m*-PBI are due to the presence of the alkyl group (octyl group). Peaks approximately 1200–1300 cm^−1^ are the breathing mode of benzimidazole and C–N stretching frequencies. The peaks at approximately 3600 cm^−1^ in *p*-PBI and pyridine-based *m*-PBI correspond to the O–H stretching frequency, which may be attributed to the presence of water in the PBI as these resins are hygroscopic and have high affinity for water.

#### 3.1.4. NMR analysis

The ^1^H and ^13^C NMR data of polymers are given in electronic supplementary material, table S2. The ^1^H NMR chemical shift values (*δ* = 13–14 ppm) indicate the presence of N–H in the polymer, and chemical shift, *δ* = 7–8 ppm, indicates C–H in the aromatic biphenyl ring protons. The chemical shift (*δ* = 110–150 ppm) observed with ^13^C NMR also clearly indicates formation of polymers. The formation of alkylated *m*-PBI was confirmed by the absence of N–H peaks in the range of 13 ppm in ^1^H NMR and also confirmed by the carbon NMR chemical shift values.

### 3.2. Batch distribution studies

**Figure 3.**
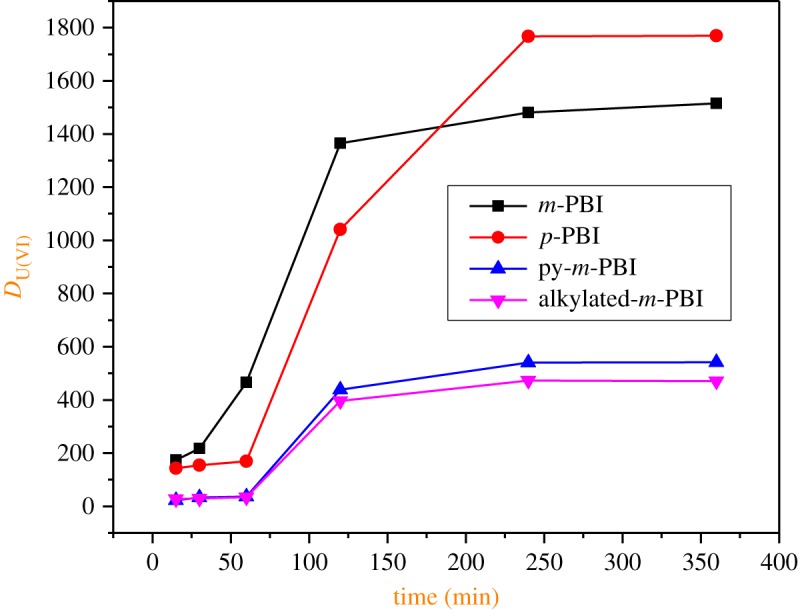
Variation of *D*_U(VI)_ as a function of time for the extraction from 0.01 M HNO_3_ solution with PBI polymeric resins.

Initially, the *D*_U(VI)_ values for the extraction from 0.01 M HNO_3_ with PBI polymers were examined as a function of equilibrium time, and the results are shown in figure 3. The *D*_U(VI)_ values were found to increase with increases in the equilibration time for all the polymers. The rate of sorption was initially slow and, after an hour, there was a steep increase in U(VI) extraction. The overall results indicate that 5 h of equilibration is adequate for the attainment of equilibrium for all the four resins. Hence, a 6 h equilibration time to ensure the attainment of equilibrium was employed for all batch extraction experiments.

**Figure 4.**
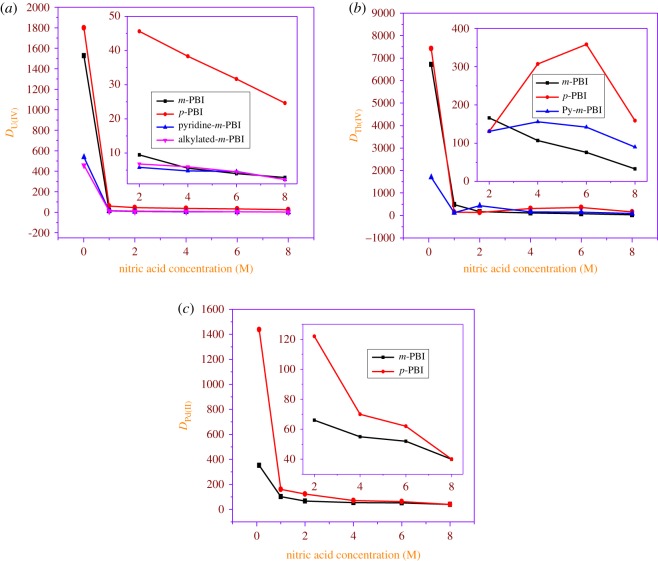
Variation of distribution ratio values as a function of HNO_3_ concentration using these polymeric resins: (*a*) variation of *D*_U(VI)_, (*b*) variation of *D*_Th(IV)_ and (*c*) variation of *D*_Pd(II)._

Figure 4*a* shows variation of *D*_U(VI)_ as a function of nitric acid concentration. It can be seen that *D-*values for the extraction of uranium decrease with increase in acidity for all four polymers. The initial steep decrease in the *D-*value with increases in the nitric acid concentration can be attributed to a cation-exchange mechanism (electronic supplementary material, scheme S1) operating in this range of acidity, involving the hydrogen bonded to nitrogen [19]. The cation-exchange mechanism was also confirmed by carrying out independent studies based on acid–base titration with phenolphthalein as the indicator. In these studies, approximately 50 mg of a polymeric resin was mixed with a known volume of 0.1 M NaOH, the contents were stirred for approximately 30 min and a few drops of phenolphthalein followed by the excess NaOH were back-titrated with standardized nitric acid solution. These studies have established that *p*-PBI is capable of exchanging metal ions through hydrogen ions. Similar studies were done with *m*-PBI and pyridine-based *m*-PBI polymeric resin. In general, all the four polymeric materials showed higher *D*_U(VI)_ values (for the extraction of uranium) from lower nitric acid medium. However, the extraction of uranium $\left(\text{U}{{\text{O}}_{2}}^{2+}\right)$ from 4 M HNO_3_ medium is mainly due to the solvation mechanism (electronic supplementary material, scheme S1), i.e. coordination to uranium through a lone pair of electrons of tertiary nitrogen present in the polymeric resin structure. It is also clear from the extraction data that *p*-PBI exhibits higher *D*_U(VI)_ values compared to other polymeric resins. The lower distribution ratios with pyridine-based *m*-PBI and alkylated *m*-PBI can be due to difficulties associated with steric effects. The U(VI) static capacity is shown in table 3.

**Table 3.** Static capacities of metal ions with PBI polymeric resins. For *p*-PBI, *m*-PBI, *py*-PBI and alkylated *m*-PBI : U(VI) feed: 600 mg l^−1^, Th(IV): 400 mg l^−1^ and Pd(II): 2000 mg l^−1^.

			static capacity (mg g^−1^ resin)		
s. no	polymeric resins	[HNO_3_] [M]	U(VI)	Th(IV)	Pd(II)
1	*m*-PBI	0.1	—	11	104
		1	11	8	90
		2	6	7	70
		4	5	6	62
		6	2	6	58
		8	2	4	46
2	*p*-PBI	0.1	50	31	182
		1	18	6	105
		2	15	6	83
		4	15	7	70
		6	13	9	68
		8	12	8	47
3	*py*-PBI	0.1	—	9	—
		1	10	8	—
		2	4	7	—
		4	5	6	—
		6	10	8	—
		8	7	7	—
4	alkylated *m*-PBI	0.1	—	—	—
		1	9	—	—
		2	4	—	—
		4	4	—	—
		6	2	—	—
		8	1	—	—

The kinetics of Th(IV) sorption (see electronic supplementary material, figure S3) was similar to sorption of uranyl ions on these PBI resins, i.e. an equilibration period of 6 h is required. Figure 4*b* shows the variation of the *D*_Th(IV)_ as a function of nitric acid concentration with PBI resins. It can be seen that *D-*values decrease with increase in acidity for all these polymeric resins. In general, all the three polymers showed higher *D*_Th(IV)_ values for the extraction of Th(IV) from lower nitric acid concentration by cation exchange (electronic supplementary material, scheme S1) compared with the 4 M HNO_3_ medium at which metal extraction is mainly by solvation (electronic supplementary material, scheme S1). It is also clear from the extraction data that *p*-PBI exhibits higher *D*_Th(IV)_ values compared with other polymers. The Th(IV) static capacity is presented in table 3.

The variations of *D*_Pd(II)_ with equilibrium nitric acid concentration for the extraction by *m*-PBI and *p*-PBI are shown in figure 4*c*. It can be seen that the *D*_Pd(II)_ value decreases with increases in nitric acid concentration of the aqueous feed. The higher *D*-values observed with Pd(II) at lower acidities compared with $\text{U}{{\text{O}}_{2}}^{2+}$ may possibly be due to operation of an anion-exchange mechanism, in addition to a typical cation-exchange mechanism (electronic supplementary material, scheme S2). The anion-exchange mechanism possibly involves protonation of a tertiary nitrogen along with a counter anion, ${{\text{NO}}_{3}}^{-}$. The palladium sorption involves the exchange of [Pd (NO_3_)_4_]^2−^ species with $\text{N}{{\text{O}}_{3}}^{-}$ ions on the polymeric resins. The extraction at higher acidity is primarily through coordination of the lone pair of electrons present on tertiary nitrogen atoms with palladium, and this mechanism plays a major role at higher acidities. Warshawsky *et al.* [44] and Wei *et al.* [45] reported a bifunctional mechanism for the extraction of palladium in their study with benzimidazole-based anion-exchange resin material. Though both these resins showed higher *D*_Pd(II)_ values for the extraction from dilute nitric acid solutions, in general, *p*-PBI exhibits higher extraction over *m*-PBI. The Pd(II) static capacity is given in table 3.

### 3.3. Removal of metal ions from aqueous medium

#### 3.3.1. Column studies with U(VI)

**Figure 5.**
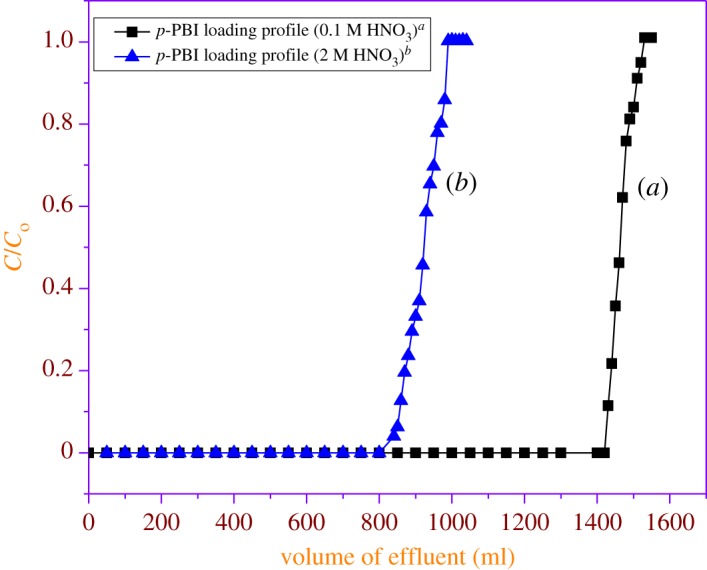
(*a*) Breakthrough behaviour for the sorption of U(VI) from a waste solution of 100 µg ml^−1^ in 0.1 M HNO_3_ with *p-*PBI polymer (flow rate: 0.5 ml min^−1^, at 298 K) from a column containing 6.5 g of *p*-PBI resin; column bed length: 6.5 cm height and 1 cm diameter and (*b*) 100 µg ml^−1^ metal in 2 M HNO_3_ with *p*-PBI (flow rate: 0.5 ml min^−1^, at 298 K). From a column containing 6.5 g of *p*-PBI resin; column bed length: 6.5 cm height and 1 cm diameter.

The dynamic capacities (breakthrough) for the columns packed with both *m*-PBI and *p*-PBI polymeric resins for the sorption of the U(VI) were determined (table 4). Under identical conditions, breakthrough capacities for the sorption of U(VI) by *p*-PBI are higher than that of *m*-PBI resin. The results on the breakthrough behaviour for the sorption of U(VI) from a solution (100 µg ml^−1^) in 0.1 M HNO_3_ with *p*-PBI polymer are shown in figure 5. Uranium loaded on the column was later eluted with a small volume of 5% ammonium carbonate solution. The eluate was analysed and found to contain 169 mg of uranium. Similarly, 1000 ml of U(VI) feed solution of 100 µg U ml^−1^ in 2 M HNO_3_ was passed through a column packed with 6.5 g of *p*-PBI (figure 5) (see electronic supplementary material, figure S9). When the uranium feed solution concentration was increased from 100 to 500 µg ml^−1^ (2 M HNO_3_), there was a fourfold decrease in the loading capacity (see electronic supplementary material, figures S5 and S6). Similarly, the U(VI) loading profile with *m*-PBI resin at 2 M nitric acid was also obtained (see electronic supplementary material, figure S4). The same resin was re-used for the second run to establish the reproducibility of performance of *p*-PBI resin.

**Table 4.** Dynamic capacities of U(VI), Th(IV) and Pd(II) on PBI polymeric resins.

s. no	polymeric resins	metal concentration in the feed (µg ml^−1^)^a^	[HNO_3_] [M]	U(VI) 100% breakthrough capacity (mg g^−1^ resin)	Th(IV) 100% breakthrough capacity (mg g^−1^ resin)	Pd(II) 100% breakthrough capacity (mg g^−1^ resin)
1	*m*-PBI	(100 µg ml^−1^)	0.1	—	10	—
		(100 µg ml^−1^)	2	6.5	—	—
2	*p*-PBI	(100 µg ml^−1^) for Th(IV), U(VI) and (200 µg ml^−1^) for Pd(II)	0.1	26	16	122
		(100 µg ml^−1^)	2	16	—	—
		(500 µg ml^−1^)	2	4.3	—	—

^a^Concentration of metal ion in the aqueous feed solution.

In the first experiment, as shown in figure 5*a*, a 100 µg ml^−1^ solution of $\text{U}{{\text{O}}_{2}}^{2+}$ from an acid (0.1 M nitric acid) was passed through the column at a flow rate of 0.5 ml min^−1^ and the 100% breakthrough profile was established. In the second experiment, as shown in figure 5*b*, 100 µg ml^−1^ solution of $\text{U}{{\text{O}}_{2}}^{2+}$ (acidity of 2 M nitric acid) was passed through the column (6.5 g of resin, 6.5 cm height and 1 cm diameter) at a flow rate of 0.5 ml min^−1^, and the 100% breakthrough profile was established. The differences in the sorption profile of $\text{U}{{\text{O}}_{2}}^{2+}$ are due to differences in sorption capacity at lower (0.1 M) and higher (2 M) acidity. This is because, at lower acidity, the $\text{U}{{\text{O}}_{2}}^{2+}$ is exchanged with N–H of *p*-PBI, whereas, at 2 M acidity, $\text{U}{{\text{O}}_{2}}^{2+}$ undergoes a solvation mechanism by coordinating to tertiary ‘N’ of the *p*-PBI resin. Hence, the capacities are different at 0.1 and 2 M for $\text{U}{{\text{O}}_{2}}^{2+}$ ions. This is also corroborated with the capacity achieved (static capacity) for $\text{U}{{\text{O}}_{2}}^{2+}$ at 0.1 M (50 mg g^−1^) and at 2 M (15 mg g^−1^) (table 3).

#### 3.3.2. Column studies with Th(IV)

**Figure 6.**
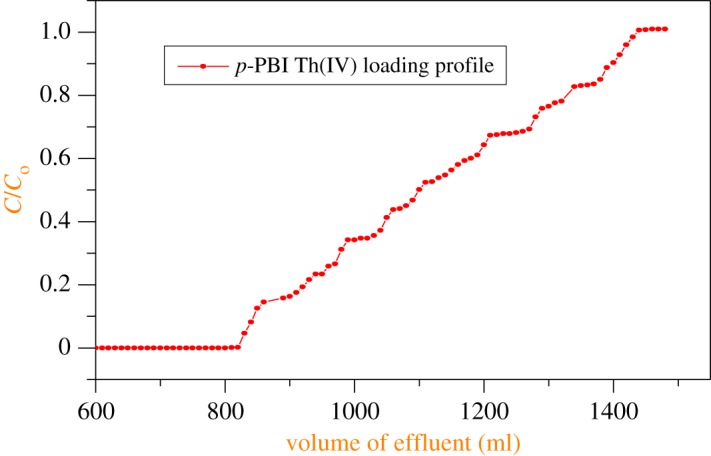
Breakthrough behaviour for the sorption of Th(IV) onto a *p*-PBI polymeric resin. Feed solution: 100 µg ml^−1^ in 0.1 M HNO_3_; flow rate: 0.5 ml min^−1^, column bed length: 6.5 cm height and 1 cm diameter. Resin quantity: 6.5 g.

Breakthrough capacities of the columns packed with both *p*-PBI and *m*-PBI polymeric resins for the sorption of Th(IV) were determined (figure 6). Approximately 1500 ml of Th(IV) solution of 100 µg ml^−1^ in 0.1 M HNO_3_ was passed through the column containing *p*-PBI. Thorium from the loaded column was subsequently eluted with a small volume of 5% sodium carbonate solution (see electronic supplementary material, figure S10). The eluate was analysed and found to contain around 104 mg of thorium. Under identical conditions, breakthrough capacities for the sorption of Th(IV) with *m*-PBI were investigated. The eluate was analysed and found to contain 65 mg of thorium. The sorption capacity of *p*-PBI is higher than that of *m*-PBI resin (see electronic supplementary material, figures S7 and S8), similar to the results obtained with uranium. Thus, *p*-PBI polymer is the candidate material for the sorption and recovery of thorium from an aqueous waste solution. Similarly, the polymer also can be employed for pre-concentration of these heavy metal ions from dilute aqueous streams. The data on the dynamic capacity of thorium with these polymers are given in table 4.

#### 3.3.3. Column studies with Pd(II)

**Figure 7.**
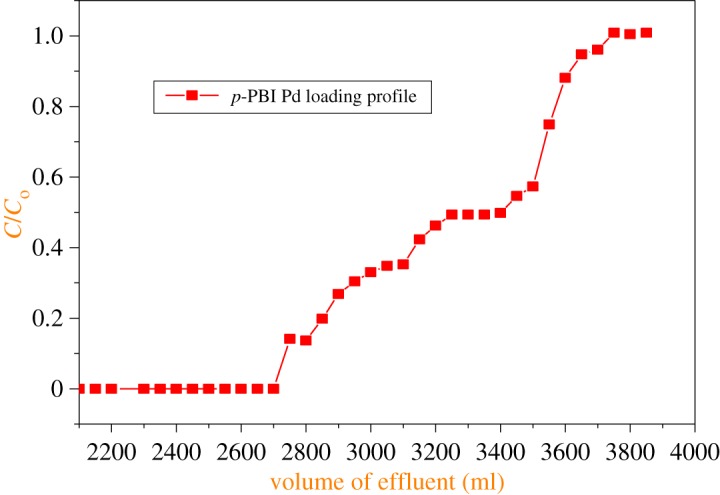
Breakthrough behaviour of Pd(II) from a simulated waste solution. Feed solution of Pd(II) of 200 µg ml^−1^ in 0.1 M HNO_3_ medium. Flow rate: 0.5 ml min^−1^, column bed length: 6.5 cm height and 1 cm width. Resin quantity: 6.5 g of *p*-PBI polymeric resin.

Figure 7 shows the loading behaviour of Pd(II) from a *p*-PBI-loaded polymeric support. Approximately 3800 ml of Pd(II) solution (200 µg ml^−1^ in 0.1 M HNO_3_) was passed through the column to attain 100% breakthrough capacity. Entire Pd(II) was subsequently eluted (732 mg) in a small volume in stripping experiments (see electronic supplementary material, figure S11); thus, *p*-PBI resin can be re-used for the removal of palladium from the aqueous stream as the sorbed metal ion species can be effectively removed in small volumes. The reusability of *p*-PBI resin was investigated by loading and stripping palladium from nitric acid medium. Three runs were conducted in these experiments and the sorption capacity was found to be constant. The dynamic capacities of palladium with *p*-PBI polymer are given in table 4.

### 3.4. Adsorption isotherm models

**Figure 8.**
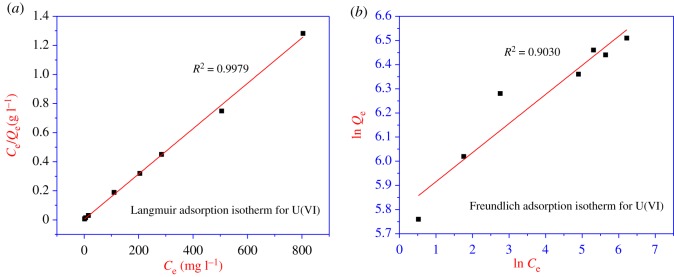
Adsorption isotherm models for U(VI)/*p*-PBI. (*a*) Langmuir adsorption isotherm and (*b*) Freundlich adsorption isotherm.

The equilibrium sorption data of U(VI) (figure 8), Th(IV) and Pd(II) on *p*-PBI in 0.1 M nitric acid medium with different feed solution concentrations, were fit into both Langmuir and Freundlich isotherm equations. The Langmuir isotherm equation provided a much better fit than the Freundlich isotherm, giving correlation coefficients greater than 0.989 in all the cases. A linear form of the Freundlich equation [33,34]:

3.1 $$\ln q_{e}=\ln K_{F}+\frac{1}{n_{F}}\ln C_{e}.$$

Therefore, the plot of ln *q*_e_ versus ln *C*_e_ gives KF as the intercept value with a slope of 1/*n*_F_. The adsorption data were analysed using the above-mentioned equation.

The linearized Langmuir isotherm equation is represented as follows [33,34]:

3.2 $$\frac{C_{e}}{q_{e}}=\frac{1}{K_{L}}+\frac{a_{L}}{K_{L}}C_{e}.$$

Hence, by plotting *C*_e_/*q*_e_ against *C*_e_, it is possible to obtain the value of *K*_L_ from the intercept, which is 1/*K*_L_ and the value of *a*_L_ from the slope, which is *a*_L_/*K*_L._ The theoretical monolayer capacity is *q*_max_ (the maximum capacity of the adsorbent) and is numerically equal to *K*_L_/*a*_L_, and this plot was found to be linear over the entire concentration range studied with a reasonably good correlation coefficient, and shows that the data fit the Langmuir adsorption isotherm than the Freundlich adsorption isotherm. The maximum monolayer saturation capacity, *q*_max_, was found to be approximately 641 mg g^−1^, approximately 168.07 mg g^−1^ and approximately 730 mg g^−1^ resin for U(VI), Th(IV) and Pd(II), respectively (table 5). The fact that the Langmuir isotherm fits the experimental data confirms the monolayer coverage of $\text{U}{{\text{O}}_{2}}^{2+}$, Th^+4^ and Pd^+2^ on the polymer pores and also the homogeneous distribution of active sites on *p*-PBI.

**Table 5.** Langmuir and Freundlich isotherm constant and linear regression coefficients.

		Freundlich isotherm			Langmuir isotherm			
s. no	sorbate/adsorbent	*K*_F_	*n*_F_	*R*^2^	*Q*_max_ (mg g^−1^)	*K*_L_ (l g^−1^)	*a*_L_ (l mg^−1^)	*R*^2^
1	UO_2_ (NO_3_)_2_/*p*-PBI	5.79	8.33	0.9030	641	301.20	0.469	0.9979
2	Th(NO_3_)_4_/*p*-PBI	7.11	5.83	0.8299	168.07	116.14	0.691	0.9983
3	Pd(NO_3_)_2_/*p*-PBI	6.14	19.04	0.7947	730	622.66	0.8530	0.9894

### 3.5. Comparison of polymers

Among the four polymers examined in the present study, *p*-PBI exhibited higher capacity for these metal ions both at lower acidity and in higher acid medium compared to other polymeric resins, *m*-PBI, pyridine-based *m*-PBI and alkylated *m*-PBI. The lower extraction ability of *m*-PBI resin, compared with the *p*-PBI polymer, is possibly due to steric reasons associated with its structure. Though alkylated *m*-polymer (–C_8_H_17_ group) is expected to display negligible extraction at lower acidity due to the absence of the N–H group, to our surprise, it has exhibited extraction for U(VI) in the lower acidity region; hence, further studies are required to understand this behaviour. In fact, the alkylated polymer was prepared through the alkylated monomer route and not by the conversion of *m*-PBI resin to the corresponding alkylated *m*-PBI resin. This is because, in the latter process, there is a possibility of some unreacted *m*-PBI component remaining in the final product. The lower extraction in the 0.1 M acidity range with pyridine-based polymer compared with *p*-PBI as well as with *m*-PBI is due to the possibility of formation of hydrogen bonds between pyridine nitrogen and imidazole hydrogen, thus reducing the hydrogen exchange capacity. In fact, in the experiments involving hydrogen ion exchange, *p*-PBI, *m*-PBI and *py*-PBI have shown exchange capacities of 3, 2.5 and 2.1 mmol, respectively.

## 4. Conclusion

Four different PBI-based polymeric resins were prepared and characterized by various techniques. The distribution ratios for the extraction of U(VI), Th(IV) and Pd(II) from dilute nitric acid medium with these polymers were determined. Among the four polymeric resins, *p*-PBI shows a higher distribution ratio for the metal ions, U(VI), Th(IV) and Pd(II). Column chromatography experiments were carried out to investigate the applications of these polymer materials (*m*-PBI and *p*-PBI) for the recovery of heavy metal ions from the simulated waste. Based on the batch extraction and column studies, *p*-PBI polymer seems to be a potential candidate for applications such as recovery of the above metal ions from mine water samples.

The recovery of heavy metal ions with PBI-based polymeric resins offers interesting opportunities to recover these metal ions, e.g. uranium from mine water samples, and other environmental waste matrices. Further studies on the influence of substituents in addressing selectivity aspects towards sorption of heavy metal ions, enhancing the shape of the beads, surface area and pore diameter, offer exciting prospects for the development of these materials for heavy metal recovery from various waste matrices.

## Supplementary Material

Electronic Supplementary Material

## Data accessibility

This article does not contain any additional data.
