# Supplementary material for: Evaluation of polybenzimidazole-based polymers for the removal of uranium, thorium and palladium from aqueous medium
Source: R Soc Open Sci. 2018 Jun 20;5(6):171701. doi: 10.1098/rsos.171701 (PMC6030331; doi:10.1098/rsos.171701)
Supplement: Electronic Supplementary Material [file rsos171701supp1.docx]

**Evaluation of Polybenzimidazole based Polymers for the Removal of Uranium, Thorium and Palladium from Aqueous Medium**

**V. Vijaya Kumar^1^, C. Ramesh Kumar^1^*, A. Suresh^2^, S. Jayalakshmi^2^, U. Kamachi Mudali^2^ and N. Sivaraman^2^**

**Electronic Supplementary Material**

**ESM Preparation 1** Alkylated monomer preparation

First step involves the alkylation of 3, 3’-dinitrobenzidine (2) [Schem 2 in main text] using the base sodium hydride in DMF. To a stirred solution of 3, 3′-dinitro,4,4′-diaminobiphenyl (2.74 g, 10 mmol) in DMF, sodium hydride (0.48 g, 20 mmol) was added at 0 ºC and the stirring was continued for about 5 minutes followed by drop-wise addition of 1-bromooctane (5.21 mL, 30 mmol). The reaction mixture was stirred for an additional 7 h at room temperature. Subsequently, the reaction mixture was quenched with cold water and extracted with dichloromethane. The organic phase was dried over anhydrous Na_2_SO_4_. The product was purified by column chromatography (5% ethyl acetate/hexane) to get alkylated-3, 3′-dinitro, 4, 4′-diaminobiphenyl (**6**), with 85% yield; melting point: 92-93 °C.

Subsequently, the nitro groups were reduced to get corresponding alkylated tetramine (7) using Sn and HCl. To a mixture of Sn metal powder (4.75 g, 40 mmol) and alkylated-3, 3′-dinitro, 4, 4′-diaminobiphenyl (4.39 g, 10 mmol), conc. HCl (50 mL) was added at 25 ºC. Then the reaction mixture was additionally stirred for 2 h at 40 ºC to ensure the completion of reduction. The hydrochloride salt of tetramine was precipitated out, which was made basic with cold 20% NaOH solution and recrystallized from hot water; the free amine was filtered out, washed with water and dried under vacuum to give alkylated- 3, 3’-diaminobenzidine (7), 88% of yield with 100% purity (see ESM Fig. 2); melting point of the compound is 94 °C.

**ESM Table 1** Data on characterization of prepared monomers by IR and NMR spectroscopy

| S.No | Compound name | Characterizations |
| --- | --- | --- |
| 1 | 3,3′-Dinitro-4,4′-diaminobiphenyl | IR (KBr, cm^-1^) 764, 819, 1095, 1188, 1250, 1298, 1353, 1408, 1463, 1511, 1551, 1638, 3084, 3173, 3364 and 3476. |
|  |  | ^1^H NMR (300 MHz, CDCl_3,_ ppm): 7.12 (d, *J* = 9 Hz, 1H); 7.56 (s, 2H); 7.75 (d, *J* = 9 Hz, 1H) and 8.14 (s, 1H). |
|  |  | ^13^C NMR (75 MHz, CDCl_3,_ ppm): 120.09, 121.25, 125.67, 130.32, 133.52 and 145.24. |
| 2 | 3, 3’-Diaminobenzidine | IR (KBr, cm^-1^) 720, 763, 822, 1088, 1165, 1277, 1410, 1502, 1575, 1634, 1876, 3018, 3050, 3185, 3302 and 3388. |
|  |  | ^1^H NMR (300 MHz, DMSO-D_6_ ppm): 4.36 (s, 2H); 4.42 (s, 2H); 6.47 (d, *J* = 8.1 Hz, 1H); 6.52 (d, *J* = 6.3 Hz, 1H) and 6.67(s, 1H). |
|  |  | ^13^C NMR: (75 MHz, DMSO-D_6,_ ppm): 112.14, 114.97, 130.92, 133.25 and 134.99. |
| 3 | 3,3′-Dinitro-4,4′-dioctylaminobiphenyl | IR (KBr, cm^-1^) 740, 760, 801, 1309, 1394, 1401, 1468, 1514, 1559, 1630, 2853, 3097 and 3362. |
|  |  | ^1^H NMR (300 MHz, CDCl_3,_ ppm): 0.91 (t, *J* = 4.8 Hz, 6H); 1.29 (m, 16H); 1.44 (m, 4H); 1.75 (sept, 4H); 3.34 (q, *J* = 6.6 Hz, 4H); 6.93 (d, *J* = 9 Hz, 2H); 7.68 (d, *J* = 9 Hz, 2H); 8.35 (s, 2H) and 8.11 (t, *J* = 4.8 Hz, 2H). |
|  |  | ^13^C NMR (75MHz, CDCl_3,_ ppm): 14.11, 22.65, 27.08, 29.01, 29.28, 31.79, 43.21, 114.66, 123.46, 126.08, 134.19 and 144.71. |
| 4 | 3,3’Dioctylaminobenzidine | IR (KBr, cm^-1^) 713, 753, 1266, 1468, 1511, 1574, 1621, 2852, 2947, 3050, 3315 and 3381. |
|  |  | ^1^H NMR (300 MHz, CDCl_3,_ ppm): 0.81 (t, *J* = 5.1 Hz, 6H); 1.22 (m, 16H); 1.33 (m, 4H); 1.59 (sept, 4H); 3.03 (t, *J* = 6.9 Hz, 4H); 3.21 (s, 4H) 6.60 (d, *J* = 9 Hz, 2H); 6.82 (s, 2H) and 6.92 (d, *J* = 6 Hz, 2H). |
|  |  | ^13^C NMR (75MHz, CDCl_3,_ ppm):14.14, 22.70, 27.35, 29.32, 29.51, 31.88, 44.47, 111.92, 114.89, 118.82, 132.21, 134.21 and 136.83. |

**ESM Table 2** Data on characterization of prepared polymers by NMR spectroscopy

| S.No | Polymer name | Characterizations |
| --- | --- | --- |
| 1 | *m*-Polybenzimidazole | ^1^H NMR (300 MHz, DMSO-D_6,_ ppm): 7.63 (s, 1H); 7.78 (s, 1H); 7.94 (d, *J* = 15 Hz, 2H), 8.15 (t, 1 H), 9.00 (d, *J* = 24 Hz, 2H), 9.34 (d, *J* = 24 Hz, 2H) and 13.69 (s, 2H). |
|  |  | ^13^C NMR (75MHz, Solid state): 113.60, 123.27, 131.09 and 146.47. |
| 2 | *p*-Polybenzimidazole | ^1^H NMR (300 MHz, DMSO-D_6,_ ppm); 7.63 (d, *J* = 9 Hz, 2H); 7.75 (d, *J* = 9 Hz, 2H); 7.94 (s, 4H); 8.02 (s, 2H) and 13.17 (s, 2H). |
|  |  | ^13^C NMR (75MHz, Solid state): 112.27, 133.22, 141.08 and 149.66. |
| 3 | Pyridine based-polybenzimidazole | ^1^H NMR (300 MHz, DMSO-D_6,_ ppm): 7.84 (d, *J* =9 Hz, 4H); 7.97 (s, 1H); 8.07 (d, *J* = 9 Hz, 2H); 8.33 (s, 2H) and 13.27 (s, 2H). |
|  |  | ^13^C NMR (75MHz, Solid state): 110.73, 119.93, 133.86, 142.54 and 149.04. |
| 4 | Alkylated m-polybenzimidazole | ^1^H NMR (300 MHz, DMSO-D_6,_ ppm): 0.79 (t, *J* = 6.6 Hz, 6H); 1.09 (m, 20H); 1.71 (sept, 4H); 4.42 (s, 4H); 7.73 (t, *J* = 8.4 Hz, 1H); 7.7 (s, 1H); 8.02 (d, *J* = 9 Hz, 2H); 8.12 (d, *J* = 9 Hz, 2H) and 8.32 (d, *J* = 6 Hz, 2H). |
|  |  | ^13^C NMR (75 MHz, DMSO-D_6,_ ppm): 13.86, 21.95, 25.75, 28.15, 28.39, 29.0, 31.03, 79.13, 111.26, 117.31, 122.35, 129.03, 129.78, 130.27, 132.64, 134.94, 135.65, 143.28 and 152.85. |
|  |  |  |

**ESM Table 3 Stripping efficiency of Uranium, Thorium and Palladium from *m*-PBI &*p*-PBI (100 mg resin sorbed with these metal ions; 5 mL stripping solution, 4-hour equilibration period for stripping)**

| **S.No** | **Metals Name** | **Stripping agent** | **% Stripping** |
| --- | --- | --- | --- |
| 1 | Uranium | 5% Ammonium Carbonate | >99 |
| 2 | Thorium | 5% Sodium Carbonate | >99 |
| 3 | Palladium | Acidic Thiourea | >99 |

**ESM Fig. 1** Retention of 3,3-Diaminobenzidine with acetonitrile as mobile phase. [Column: C_18_ (250 X 4.6mm, 5µ, Hypersil)], Mobile phase: Acetonitrile, Flow rate: 1mL/min. Detection: 254 nm.

**ESM Fig. 2** Retention of 3,3-Dioctylaminobenzidine with acetonitrile as mobile phase. [Column: C_18_ (250X4.6mm, 5µ, Hypersil)], Mobile phase: Acetonitrile, Flow rate: 1mL/min. Detection 254 nm.

**ESM Fig. 3** Variation in the *D*_Th(IV)_ with time at 0.1 M HNO_3_ with PBI polymeric resins

**ESM Fig. 4** Breakthrough behaviour of U(VI) from simulated waste solution. Feed solution of U(VI) containing (100µg/mL) from 2 M HNO_3_ medium. Flow rate: 0.5 mL/min, column bed length: 6.5 cm height and 1 cm dia. Resin quantity: 6.5 g of *m*-PBI polymeric resin.

**ESM Fig. 5** Breakthrough behaviour of U(VI) from feed solution of U(VI) containing (500µg/mL) from 2 M HNO_3_ medium. Flow rate: 0.5 mL/min, column bed length: 6.5 cm height and 1 cm dia. Resin quantity: 6.5 g of *p*-PBI polymeric resin.

**ESM Fig. 6** Elution profile of uranium from *m*-PBI loaded column (polymeric resin sorbed with uranium) Eluting solution: 5% ammonium carbonate; resin bed volume: 10 mL, flow rate: 0.5 ml/min, (ESM Fig.5. Elution profile)

**ESM Fig. 7** Breakthrough behaviour for the sorption of Th(IV) from a feed solution containing 100 μg /mL metal in 0.1M HNO_3_ with *m*-PBI polymeric resin; feed solution flow rate: 0.5 mL/min, column bed length: 6.5 cm height and 1 cm dia. Resin quantity: 6.5 g of *m*-PBI polymeric resin.

**ESM Fig. 8** Elution profile for Th(IV) loaded *m*-PBI polymeric resin packed column. Eluting solution 6% sodium carbonate; resin bed volume: 10 mL, flow rate: 0.5 mL/min. (ESM, Fig.8. Elution profile)

**ESM Fig. 9** Elution profile of uranyl ion from a *p*-PBI polymeric supported column. Mobile phase: 5% ammonium carbonate for elution of U(VI); flow rate: 0.5 mL/min.

**ESM Fig. 10** Elution profile for Th(IV) from *p*-PBI. Mobile phase: 6% sodium carbonate solution in water. Resin bed volume: 10 mL; 6.5 g resin, Flow rate-0.5 ml/min.

**ESM Fig. 11** Elution profile for Pd(II) loaded column (Eluting solution 1 M thiourea in 0.1 M HNO_3_) *p*-PBI, Resin bed volume: 10 mL, Flow rate: 0.5 mL/min.

|  |
| --- |

**ESM Scheme 1** The proposed cation exchange and solvation mechanism of uranium and thorium.

|  |
| --- |

**ESM Scheme 2** The possible cation and anion exchange mechanism for palladium.
